# Supplementary material for: Mesopelagic microbial community dynamics in response to increasing oil and Corexit 9500 concentrations
Source: PLoS One. 2022 Feb 23;17(2):e0263420. doi: 10.1371/journal.pone.0263420 (PMC8865645; doi:10.1371/journal.pone.0263420)
Supplement: S1 Fig — Created in R using vector map data from Natural Earth, free vector and raster map data @ naturalearthdata.com. All map data from Natural Earth is in the public domain and free for use for any personal, educational, or commercial purpose. (DOCX) [file pone.0263420.s001.docx]

**Figure S1.** Sampling site. Created in R using vector map data from Natural Earth, free vector and raster map data @ naturalearthdata.com. All map data from Natural Earth is in the public domain and free for use for any personal, educational, or commercial purpose.
