# Supplementary material for: Mesopelagic microbial community dynamics in response to increasing oil and Corexit 9500 concentrations
Source: PLoS One. 2022 Feb 23;17(2):e0263420. doi: 10.1371/journal.pone.0263420 (PMC8865645; doi:10.1371/journal.pone.0263420)
Supplement: S4 Fig — Number of aromatic rings are shown on the upper panel. Note the different y-axis scales for each panel. (DOCX) [file pone.0263420.s004.docx]

**Figure S4.** Mean concentrations of 19 residual PAHs and 22 groups of alkyl-PAHs of oil containing treatments (WAF and all 3 concentrations of CEWAF). Number of aromatic rings are shown on the upper panel. Note the different y-axis scales for each panel.
