# Supplementary material for: Mesopelagic microbial community dynamics in response to increasing oil and Corexit 9500 concentrations
Source: PLoS One. 2022 Feb 23;17(2):e0263420. doi: 10.1371/journal.pone.0263420 (PMC8865645; doi:10.1371/journal.pone.0263420)
Supplement: S6 Fig — (DOCX) [file pone.0263420.s006.docx]

**Figure S6.** Rarefaction curves of each treatment during the 6-week incubation period.
