# Supplementary material for: Mesopelagic microbial community dynamics in response to increasing oil and Corexit 9500 concentrations
Source: PLoS One. 2022 Feb 23;17(2):e0263420. doi: 10.1371/journal.pone.0263420 (PMC8865645; doi:10.1371/journal.pone.0263420)
Supplement: S7 Fig — The samples are color-coordinated according to treatment, while the different shapes represent incubation time. (DOCX) [file pone.0263420.s007.docx]

**Figure S7.** Principal coordinates analysis (PCoA) of bacterial community composition within each treatment during the 6-week experiment based on Bray-Curtis dissimilarities. The samples are color-coordinated according to treatment, while the different shapes represent incubation time.
