# Supplementary material for: Mesopelagic microbial community dynamics in response to increasing oil and Corexit 9500 concentrations
Source: PLoS One. 2022 Feb 23;17(2):e0263420. doi: 10.1371/journal.pone.0263420 (PMC8865645; doi:10.1371/journal.pone.0263420)
Supplement: S9 Fig — Analysis of similarities (ANOSIM) plot showing dissimilarity with R and P values based between and within A) Time Points, B) Treatments. Bold horizontal bar in box indicates median; bottom of box indicates 25th percentile; top of box indicates 75th percentile; whiskers extend to the most extreme data point; width of bar is directly proportional to sample size. (DOCX) [file pone.0263420.s009.docx]

**Figure S9.** Analysis of similarities (ANOSIM) plot showing dissimilarity with R and P values based between and within A) Time Points, B) Treatments. Bold horizontal bar in box indicates median; bottom of box indicates 25th percentile; top of box indicates 75th percentile; whiskers extend to the most extreme data point; width of bar is directly proportional to sample size.
