# Supplementary material for: Mesopelagic microbial community dynamics in response to increasing oil and Corexit 9500 concentrations
Source: PLoS One. 2022 Feb 23;17(2):e0263420. doi: 10.1371/journal.pone.0263420 (PMC8865645; doi:10.1371/journal.pone.0263420)
Supplement: S10 Fig — Each bar is the average of triplicate treatment. (DOCX) [file pone.0263420.s010.docx]

Figure S10. Relative abundances of top 15 microbial genera observed *in-situ* seawater samples and each treatment over the 6-week experiment. Each bar is the average of triplicate treatment.
