# Supplementary material for: Mesopelagic microbial community dynamics in response to increasing oil and Corexit 9500 concentrations
Source: PLoS One. 2022 Feb 23;17(2):e0263420. doi: 10.1371/journal.pone.0263420 (PMC8865645; doi:10.1371/journal.pone.0263420)
Supplement: S12 Fig — Each bar represents the average of triplicate treatments (DOCX) [file pone.0263420.s012.docx]

**Figure S12.** Relative abundances of selected ASVs belonging to *Alteromonas.* Each bar represents the average of triplicate treatments
