# Supplementary material for: Mesopelagic microbial community dynamics in response to increasing oil and Corexit 9500 concentrations
Source: PLoS One. 2022 Feb 23;17(2):e0263420. doi: 10.1371/journal.pone.0263420 (PMC8865645; doi:10.1371/journal.pone.0263420)
Supplement: S1 Table — (DOCX) [file pone.0263420.s013.docx]

Table S1. Number of sequence reads filtered through each step of the DADA2 pipeline.

|  | **Input** | **Filtered** | **Denoised R1** | **Denoised R2** | **Merged** | **Non-Chimeras** |
| --- | --- | --- | --- | --- | --- | --- |
| **API-Blank** | 386 | 341 | 330 | 334 | 322 | 322 |
| ***In-situ* seawater** | 6386 | 3994 | 3388 | 3660 | 2917 | 2917 |
| **T0-CEWAF-High-A** | 76286 | 71918 | 71426 | 71579 | 69016 | 68270 |
| **T0-CEWAF-High-B** | 82657 | 76164 | 75936 | 75958 | 72943 | 72701 |
| **T0-CEWAF-High-C** | 127884 | 119854 | 119101 | 119288 | 114740 | 112598 |
| **T0-CEWAF-Low-A** | 71228 | 67834 | 66925 | 67272 | 63741 | 63457 |
| **T0-CEWAF-Low-B** | 94575 | 88720 | 87779 | 87966 | 83096 | 82592 |
| **T0-CEWAF-Low-C** | 88556 | 83603 | 82549 | 82910 | 77989 | 77255 |
| **T0-CEWAF-Med-A** | 89232 | 83798 | 82723 | 83000 | 78015 | 77569 |
| **T0-CEWAF-Med-B** | 98143 | 92960 | 91832 | 92124 | 87045 | 86609 |
| **T0-CEWAF-Med-C** | 80394 | 76770 | 75654 | 76026 | 72687 | 72230 |
| **T0-Corexit-High-A** | 92179 | 83871 | 83553 | 83735 | 80872 | 80840 |
| **T0-Corexit-High-B** | 63110 | 59555 | 59356 | 59378 | 58071 | 57860 |
| **T0-Corexit-High-C** | 43238 | 40564 | 40480 | 40491 | 39533 | 38995 |
| **T0-Corexit-Low-A** | 129905 | 122405 | 121333 | 121692 | 115927 | 115251 |
| **T0-Corexit-Low-B** | 79798 | 75225 | 74658 | 74862 | 71113 | 71111 |
| **T0-Corexit-Low-C** | 80398 | 74459 | 73725 | 74036 | 70826 | 70570 |
| **T0-Corexit-Med-A** | 99022 | 94579 | 93885 | 93986 | 90844 | 90473 |
| **T0-Corexit-Med-B** | 74935 | 70943 | 70299 | 70480 | 67607 | 67378 |
| **T0-Corexit-Med-C** | 63516 | 60131 | 59620 | 59763 | 57569 | 57377 |
| **T0-SW-A** | 55616 | 53457 | 52515 | 52814 | 49976 | 49717 |
| **T0-SW-B** | 58152 | 55818 | 55026 | 55276 | 52523 | 52418 |
| **T0-SW-C** | 129184 | 120040 | 118767 | 119210 | 112075 | 111558 |
| **T0-WAF-A** | 58064 | 56154 | 55078 | 55338 | 52315 | 51866 |
| **T0-WAF-B** | 105567 | 99352 | 97831 | 98210 | 92479 | 91164 |
| **T0-WAF-C** | 85370 | 78623 | 77384 | 77697 | 72919 | 72389 |
| **T3-CEWAF-High-A** | 160048 | 151125 | 150176 | 150679 | 144513 | 112457 |
| **T3-CEWAF-High-B** | 170067 | 161894 | 160963 | 161300 | 156047 | 132698 |
| **T3-CEWAF-High-C** | 113458 | 106837 | 106197 | 106511 | 103509 | 93171 |
| **T3-CEWAF-Low-A** | 154577 | 143719 | 142371 | 142945 | 132400 | 107303 |
| **T3-CEWAF-Low-B** | 202693 | 190600 | 189190 | 189852 | 182309 | 156717 |
| **T3-CEWAF-Low-C** | 6 | 3 | 1 | 1 | 0 | 0 |
| **T3-CEWAF-Med-A** | 105398 | 99582 | 98885 | 99267 | 95456 | 85440 |
| **T3-CEWAF-Med-B** | 6 | 3 | 1 | 1 | 0 | 0 |
| **T3-CEWAF-Med-C** | 5 | 2 | 1 | 1 | 1 | 1 |
| **T3-Corexit-High-A** | 130143 | 120146 | 119718 | 119664 | 113797 | 107762 |
| **T3-Corexit-High-B** | 135230 | 127235 | 126728 | 126827 | 122308 | 114983 |
| **T3-Corexit-High-C** | 143555 | 134124 | 133542 | 133704 | 128992 | 122047 |
| **T3-Corexit-Low-A** | 166089 | 152172 | 151024 | 151524 | 141173 | 117398 |
| **T3-Corexit-Low-B** | 80220 | 77446 | 76390 | 76836 | 70964 | 55600 |
| **T3-Corexit-Low-C** | 62958 | 59997 | 58847 | 59538 | 53973 | 36977 |
| **T3-Corexit-Med-A** | 114088 | 107230 | 106606 | 106815 | 102166 | 88589 |
| **T3-Corexit-Med-B** | 147029 | 138546 | 137790 | 138091 | 131262 | 108702 |
| **T3-Corexit-Med-C** | 183174 | 171654 | 170780 | 171026 | 161411 | 134294 |
| **T3-SW-A** | 121925 | 115671 | 114851 | 115188 | 111784 | 106215 |
| **T3-SW-B** | 3 | 3 | 1 | 1 | 0 | 0 |
| **T3-SW-C** | 100495 | 94996 | 94142 | 94448 | 91297 | 86870 |
| **T3-WAF-A** | 143532 | 135124 | 134461 | 134720 | 129795 | 118280 |
| **T3-WAF-B** | 179951 | 168836 | 167928 | 168347 | 159056 | 132447 |
| **T6-CEWAF-High-A** | 133723 | 125485 | 124526 | 125078 | 118579 | 98022 |
| **T6-CEWAF-High-B** | 107252 | 102009 | 101301 | 101720 | 98256 | 85313 |
| **T6-CEWAF-High-C** | 126315 | 119897 | 119047 | 119208 | 114122 | 95520 |
| **T6-CEWAF-Low-A** | 102853 | 96018 | 95149 | 95599 | 91406 | 79475 |
| **T6-CEWAF-Low-B** | 142018 | 133718 | 132523 | 133070 | 124884 | 98874 |
| **T6-CEWAF-Low-C** | 118787 | 111323 | 110393 | 110831 | 105843 | 92000 |
| **T6-CEWAF-Med-A** | 211413 | 197420 | 196169 | 196709 | 185264 | 149814 |
| **T6-CEWAF-Med-B** | 139533 | 131212 | 130389 | 130761 | 124444 | 108541 |
| **T6-CEWAF-Med-C** | 63003 | 60329 | 59932 | 60177 | 58428 | 52443 |
| **T6-Corexit-High-A** | 37292 | 36436 | 36148 | 36315 | 35456 | 32368 |
| **T6-Corexit-High-B** | 134012 | 123997 | 123454 | 123593 | 118472 | 110855 |
| **T6-Corexit-High-C** | 111052 | 104937 | 104462 | 104629 | 101678 | 95454 |
| **T6-Corexit-Low-A** | 127040 | 120566 | 119280 | 120055 | 111353 | 82741 |
| **T6-Corexit-Low-B** | 116686 | 109933 | 109031 | 109420 | 103389 | 86748 |
| **T6-Corexit-Low-C** | 104655 | 97873 | 97055 | 97540 | 93025 | 77326 |
| **T6-Corexit-Med-A** | 117136 | 109594 | 108967 | 109264 | 104383 | 88758 |
| **T6-Corexit-Med-B** | 157253 | 146363 | 145570 | 145985 | 139406 | 123808 |
| **T6-Corexit-Med-C** | 132425 | 123978 | 123263 | 123578 | 116927 | 101320 |
| **T6-SW-A** | 117681 | 112776 | 112056 | 112368 | 109445 | 93264 |
| **T6-SW-B** | 146094 | 134965 | 134147 | 134526 | 128324 | 108693 |
| **T6-SW-C** | 210767 | 199325 | 198387 | 198737 | 192106 | 165093 |
| **T6-WAF-A** | 103466 | 97153 | 96304 | 96714 | 92569 | 79801 |
| **T6-WAF-B** | 121415 | 114216 | 113411 | 113859 | 108902 | 87661 |
| **T6-WAF-C** | 146636 | 136840 | 135983 | 136407 | 129931 | 113629 |
