# Supplementary material for: Mesopelagic microbial community dynamics in response to increasing oil and Corexit 9500 concentrations
Source: PLoS One. 2022 Feb 23;17(2):e0263420. doi: 10.1371/journal.pone.0263420 (PMC8865645; doi:10.1371/journal.pone.0263420)
Supplement: Attachment — Submitted filename: Aljandal_dosage_ReviewerResponses_24nov21_FINAL.pdf [file pone.0263420.s014.pdf]

Response to Reviewers for:

Mesopelagic Microbial Community Dynamics in Response to Increasing Oil and Corexit 9500 Concentrations

Shahd Aljandal, Shawn M Doyle, Gopal Bera, Terry L. Wade, Anthony H. Knap and Jason B. Sylvan

Reviewer #1: In the manuscript " Mesopelagic Microbial Community Dynamics in Response to Increasing Oil and Corexit 9500 Concentrations", the authors reported the response of natural marine microbial community from the Gulf of Mexico to different concentrations of chemically dispersed crude oil. The authors further analysed the alkane and PAHs concentration dynamics in the different treatments. Although the manuscript is well written and the experiments are sound with good validation, there are two main issues: (1) what are the advantages or innovations of this study compared to previous reported studies apart from the fact that the authors used three different concentrations of dispersants? It would have been more intriguing if multiple dispersants were compared or more advanced ecological analyses were performed to better understand the effects on the microbial populations; and (2) I'm not sure that the results really change much in terms of our view of how oil degradation occurs, and how to make the processes more efficient. There are no surprises that might change the current paradigm.

> *Author response: We appreciate the reviewer's comments and address the more specific ones below. In regard to advantages of this study compared to previous, as pointed out in the introduction and discussion, prior dosage experiments are limited to surface waters and chemical analysis only, really in just one paper (Prince et al., 2017). We here sought to conduct a dosage experiment and analyze microbial response over weeks to months, not just hydrocarbon chemistry. Additionally, this is the first such experiment from mesopelagic waters. We do think that a comparison of different dispersants is interesting and enjoyed the recent paper published by Dr. Nikolova in Microbiome, but that was not the intent of this experiment.*
>
> *While the results do not shift paradigms concerning microbial hydrocarbon degradation, we believe they are unique and informative. A mission statement of PLoS One is that the journal* evaluates *"research on scientific validity, strong methodology, and high ethical standards—not perceived significance." Therefore, our understanding is that submitted manuscripts do not need to be paradigm changing to be publishable.*

1. What protocol was used for the preparation of WAF and CEWAF? The mixing speed seems too high (e.g. see Aurand, D. and G. Coelho (Editors). 2005. Cooperative Aquatic Toxicity Testing of Dispersed Oil and the "Chemical Response to Oil Spills: Ecological Effects Research

Forum (CROSERF)." Ecosystem Management & Associates, Inc. Lusby, MD. Technical Report 07-03, 105 pages + Appendices).

Author response: The WAF preparation section in the Methods has been updated to include a reference to Kleindiesnt et al., from which we based our methods. We also added specific mention of settling time for CEWAF. The CROSERF method is a non-vortex method but could never achieve the high concentrations observed by Kleindiesnt and we wanted to see the biological response of a high concentration of WAF.

2. It seems like there was no head space left in the microcosm bottles and therefore, how did the authors ensure that there was adequate oxygenation of the microcosms?

Author response: We realized that the photo originally shown in Figure S2 could be confusing (removed in the current version) - what was shown in that photo was the WAF preparation process, not the actual experiment setting. In the experiment bottles, there was about 500 mL head space in each bottle.

3. How did the authors mix the bottles for the duration of the experiment, and if not, how did they avoid the settling of the crude oil on the water surface?

Author response: We did not mix, but because we used WAF in our experiments, there was no layer of crude oil at the surface. Again, this misconception may be due to the photo from the original version of Figure S2, which has been removed.

4. The authors analysed the concentrations of DOSS but no further discussion is provided of why this was done and what are the implications for the microbial community.

Author response: The reviewer makes a good point. The DOSS data was not critical to our analysis and we therefore removed it to make the manuscript clearer and more streamlined.

In addition, there are a few minor revisions that could be addressed to improve the manuscript.

Line 93: Why was the field water kept at 10C? I assume that this was the temperature of the seawater at the time of collection?

Author response: This was in situ *temperature of the water when collected. We now indicate this on Line 93.*

Line 100: What is the rationale for using these concentrations? Please, give more context.

Author response: Good question - we added rationale on lines 106-110. Briefly, the medium concentration is realistic for in situ values during the Deepwater Horizon spill, and one order of magnitude above and below is likely to elicit a response that allows us to determine patterns based on dosage.

Line 101: Are the low, medium and high concentrations in the Corexit alone treatments the same as in the CEWAF treatments? The supplementary information suggest otherwise but that needs to be explained better.

Author response: Thank you for pointing this out, the concentrations are the same and we have modified the text to better explain this (Lines 103-106).

Line 105: There should be space between “1.4” and “L”

Author response: The suggested change has been made.

Line 112: Please, specify the duration of the experiment here. You have mentioned it further down but i think this needs to be mentioned in the methods section so that the reader does not end up scrolling up and down to find this information**.** In addition, three weeks seems a lot of time between sampling points. Previous studies have shown that microbial degradation starts very early on.

Author response: We added the experimental duration here (now line 121). We acknowledge that changes in microbiology and chemistry do occur prior to 3 weeks, as indicated in prior experiments from this lab, but the focus here was on longer term changes and comparisons between dosing percent.

Line 115: Can you specify the exact volume of CEWAF and Dispersant only in the treatments?

Author response - We have included more specific mention of volume of sample for these treatments (Lines 124-127).

Line 160: Add space between “5” and “ul”.

Author response: The suggested change has been made.

Line 163: Add space between “2.1” and “mm”, “100” and “mm”, and “1.8” and “um”.

Author response: The suggested change has been made.

Line 190-191: Specify the barcoded primers used for the 16S rRNA amplification.

Author response: Good point! It was 515F and 806R, we now indicate this on line 163.

Line 177: Add space between “25” and “mm”

Author response: The suggested change has been made.

Line 199: The full stop should be after the citation (33).

Author response: The suggested change has been made.

Line 223: The “n” in front of C14-C22 should be in Italics. Please, amend the rest of these occurrences in the rest fo the text.

Author response: The suggested change has been made.

Figure 1. See my previous comment re n-C17, n-C18. Make these consistent throughout the text.

Author response: The suggested change has been made.

Figure 2: How would you explain the higher number of cells in the SW control at T6 than in the WAF at T6?

Author response: We are not totally sure why cell counts are higher in SW control compared to WAF at T6, it is a 2.5-fold difference. We are comfortable assuming this is natural biological variability and do not think it warrants discussion in the text. For reference, we have seen this in previous incubation experiments (Doyle et al., 2018, Frontiers in Microbiology; *Doyle et al., 2020,* mSystems*).*

Line 268: Delete “oil” in the sentence “…micro-aggregates in oiled treatments oil after three weeks”.

Author response: The suggested change has been made.

Line 280: It would have been useful to compare the Bray-Curtis PCoA with for example weighted UniFrac as the phylogenetic distance might provide better explanation for the variation between treatments.

Author response: The suggested change has been made. Both methods showed the same conclusion; we now include the UniFrac analysis in the main text (Figure 3) and the Bray-curtis analysis in the supplement (Figure S8).

Specify in the methods or results how many paired-end sequences were obtained per sample on average and what was the SD between them. What is the number of ASVs after filtration.

Author response: The suggested change has been made. We added Table S1, which has this data, and refer to it in the Methods section under "Molecular Biology, DNA Sequencing and Analysis." (line 188)

Figure 4: It would be more helpful if the figure showed the taxonomic units at family level or show top 15 or 25 genera. Did the authors obtained a taxonomic profile of the in-situ seawater at the time of sampling? This information could be quite useful to better understand how the indigenous community changed when oil/dispersant was added.

Author response: We revised Figure 3 to include all microbes on the order level, Figure 5 already has hydrocarbon degraders on ASV/Genera level as suggested. We also included the suggested figure (fig. S10) to include top 15 genera of Figure 3. Figure 3 was revised to include the taxonomic profile of the in-situ seawater sample at the time of sampling.
